# The Influence of Omega-3 Fatty Acids on Skeletal Muscle Protein Turnover in Health, Disuse, and Disease

**DOI:** 10.3389/fnut.2019.00144

**Published:** 2019-09-06

**Authors:** Chris McGlory, Philip C. Calder, Everson A. Nunes

**Affiliations:** ^1^School of Kinesiology and Health Studies, Queen's University, Kingston, ON, Canada; ^2^Human Development and Health, Faculty of Medicine, University of Southampton, Southampton, United Kingdom; ^3^NIHR Southampton Biomedical Research Centre, University Hospital Southampton NHS Foundation Trust and University of Southampton, Southampton, United Kingdom; ^4^Department of Physiological Sciences, Federal University of Santa Catarina, Florianópolis, Brazil

**Keywords:** Omega-3 fatty acid, protein synthesis, protein breakdown, skeletal muscle, inflammation

## Abstract

Ingestion of omega-3 fatty acids is known to exert favorable health effects on a number of biological processes such as improved immune profile, enhanced cognition, and optimized neuromuscular function. Recently, data have emerged demonstrating a positive influence of omega-3 fatty acid intake on skeletal muscle. For instance, there are reports of clinically-relevant gains in muscle size and strength in healthy older persons with omega-3 fatty acid intake as well as evidence that omega-3 fatty acid ingestion alleviates the loss of muscle mass and prevents decrements in mitochondrial respiration during periods of muscle-disuse. Cancer cachexia that is characterized by a rapid involuntary loss of lean mass may also be attenuated by omega-3 fatty acid provision. The primary means by which omega-3 fatty acids positively impact skeletal muscle mass is via incorporation of eicosapentaenoic acid (EPA; 20:5*n*−3) and docosahexaenoic acid (DHA; 22:6*n*−3) into membrane phospholipids of the sarcolemma and intracellular organelles. Enrichment of EPA and DHA in these membrane phospholipids is linked to enhanced rates of muscle protein synthesis, decreased expression of factors that regulate muscle protein breakdown, and improved mitochondrial respiration kinetics. However, exactly how incorporation of EPA and DHA into phospholipid membranes alters these processes remains unknown. In this review, we discuss the interaction between omega-3 fatty acid ingestion and skeletal muscle protein turnover in response to nutrient provision in younger and older adults. Additionally, we examine the role of omega-3 fatty acid supplementation in protecting muscle loss during muscle-disuse and in cancer cachexia, and critically evaluate the molecular mechanisms that underpin the phenotypic changes observed in skeletal muscle with omega-3 fatty acid intake.

## Introduction

Omega-3 (n-3) polyunsaturated fatty acids are a class of long chain fatty acids reported to have a range of beneficial effects on human health such as improved immune profile, enhanced cognition, blood lipid regulation, and optimized neuromuscular function (1–3). The beneficial impact of omega-3 fatty acid ingestion on health markers is often related to increases in the omega-3 fatty acid content of phospholipids in membranes at the expense of omega-6 fatty acids (1). This shift in the omega-3: omega-6 fatty acid ratio in cell membranes has been shown to induce changes in a multitude of biological processes including the expression of pro- and anti-inflammatory lipid mediators and cytokines (1), gene expression (4), and mitochondrial respiration kinetics (5, 6). As dysregulation of these processes is closely linked with impaired metabolic health (1, 7), omega-3 fatty acid intake could be considered a viable strategy to combat metabolic dysfunction in a variety of settings.

Eicosapentaenoic acid (EPA; 20:5*n*−3) and docosahexaenoic acid (DHA; 22:6*n*−3) are the most studied omega-3 fatty acids and can be found in oily fish and many dietary supplements. EPA and DHA serve as the necessary substrates for the production of anti-inflammatory and inflammation resolving mediators (resolvins, protectins, and maresins) whilst simultaneously inhibiting the transcription of pro-inflammatory genes (1, 4). Current population recommendations for EPA and DHA intake for general health vary from country to country but are typically 250–500 mg/day as a combination of both fatty acids (8). Although humans can endogenously synthesize EPA and DHA from dietary alpha linolenic acid they are considered conditionally essential as the synthesis of EPA and DHA from alpha linolenic acid is limited in humans. Indeed, the conversion of alpha linolenic acid to EPA and DHA in men is estimated to be as low as <8% and <4% respectively, whilst in women it is slightly higher at 21 and 9%, respectively (9). Therefore, dietary or supplemental intake of preformed EPA and DHA intake is necessary to significantly enhance the EPA and DHA content of biological tissues in humans with known significant inter-individual variability (10).

In recent years, there have been a number of reports in cell systems (11, 12), pre-clinical mammalian models (13–15) as well as humans (16–18) demonstrating a positive influence of EPA and DHA intake on skeletal muscle. The notion that EPA and DHA intake may affect skeletal muscle has garnered much attention not only because skeletal muscle mass and strength are important in promoting metabolic health (19) and longevity (20), but are also critical determinants of recovery from situations of accelerated muscle loss (e.g., surgery/intensive care) (21). Yet, the underlying mechanisms by which EPA and DHA intake confer a positive effect on skeletal muscle remain unclear. In this review, we address data related to the interaction between omega-3 fatty acid ingestion and skeletal muscle anabolism with a specific emphasis on muscle protein turnover kinetics and translational control. Additionally, we examine the potential efficacy of omega-3 fatty acid supplementation to counteract muscle loss during periods of muscle-disuse, cancer cachexia, as well as the relevance of inflammatory signaling events. Given the focused nature of this review we apologize in advance to respected colleagues whose work we were unable to address and instead refer the interested reader to excellent commentaries on this topic (22–24).

### Regulation of Skeletal Muscle Mass

In healthy, normal-weight individuals, skeletal muscle comprises ~45% of body mass and plays a fundamental role in locomotion, respiration, amino acid storage, glycemic control, and the ability to sustain independent living with aging (19). Understanding the factors that regulate skeletal muscle mass is therefore critical for the development of strategies to support optimal health across the lifespan. The size and composition of skeletal muscle is determined by changes in rates of muscle protein synthesis (MPS) relative to those of muscle protein breakdown. In the rested, fasted-state, the rate of MPS is lower than that of muscle protein breakdown resulting in a negative state of protein balance (25). The ingestion of high-quality protein, rich in essential amino acids stimulates a transient increase in the rate of MPS resulting in a positive state of muscle protein balance (26, 27). It is also known that a single bout of resistance exercise performed in the fasted-state will induce a rise in both MPS and breakdown; however, the rate of MPS is elevated 48 h post-exercise whereas the rate of breakdown is returned to baseline at 48 h post-exercise (28). Critically, protein feeding and resistance exercise impart additive effects on MPS and net protein balance (26, 29) so when repeated bouts of resistance exercise are coupled with adequate protein feeding there is a protracted state of positive muscle protein balance leading to a gradual increase in skeletal muscle size (30).

### Omega-3 Fatty Acids and Skeletal Muscle Lipid Profiles

Omega-3 fatty acid status is often assessed using either venous or fingerpick blood samples followed by analysis for fatty acid composition of membrane phospholipids with a number of basic mathematical calculations based on the relative abundance of omega-3 fatty acids to other fatty acids employed to determine risk of either disease or deficiency (31, 32). Changes in the omega-3 fatty acid composition of human blood membrane phospholipids with omega-3 fatty acid intake occurs rapidly (days) in a dose-dependent manner (33, 34) with washout kinetics exhibiting comparable declines following the cessation of intake (35). Due to slower turnover rates compared to blood, changes in omega-3 fatty acid composition of whole skeletal muscle phospholipid profiles even with high doses of omega-3 fatty acid intake (~3 g/d EPA and ~2 g/d DHA) require at least 2 weeks of supplementation in young men before detectable changes are observed (33). Recent work in young women using a similar dose of EPA + DHA has also demonstrated increases in the omega-3 fatty acid content of skeletal muscle phospholipids that plateaued somewhere between 6 and 8 weeks of supplementation (36). Interestingly, work in human blood has shown that there are sex-dependent effects in the change in the ratio of EPA:DHA with omega-3 fatty acid supplementation (35); however, no study has directly compared changes in omega-3 fatty acid skeletal muscle phospholipid profiles between men and women with omega-3 fatty acid intake. Moreover, unlike blood (35), no study has established a dose-response and washout of skeletal muscle phospholipid omega-3 fatty acid content with omega-3 fatty acid supplementation. Another important consideration is that the rate of incorporation of omega-3 fatty acids into skeletal muscle phospholipid membranes may differ depending on the fraction assessed (e.g., whole muscle vs. sarcolemmal vs. mitochondrial) (37). As both the sarcolemmal and mitochondrial membranes serve as major sites of protein interactions and substrate transport, understanding how omega-3 fatty acid intake alters the lipid composition of distinct cellular organelles would provide key insights into the impact of compartmental lipid shifts on skeletal muscle physiology.

## Omega 3 Fatty Acids and Muscle Protein Turnover

One of the first reports suggesting that omega-3 fatty acid intake alters muscle protein turnover *in vivo* was conducted in growing steers. In that study, Gingras et al. (38) examined the impact of omega-3 fatty acid-enriched menhaden oil infusion (13.5% EPA and 14.4% DHA) on whole-body protein kinetics using isotopically-labeled phenylalanine coupled with infusion of amino acids and insulin. The primary finding was that following omega-3 fatty acid provision, there was a doubling in the amount of amino acids required to prevent a state of hypo-aminoacidemia during a hyper-insulinemic clamp; indicative of increased whole-body protein anabolism. The authors speculated that the increased rate of amino acid clearance from the systemic circulation following omega-3 fatty acid provision was likely a function of either greater amino acid uptake into peripheral tissues, increased amino acid oxidation, and/or a reduction in the rate of protein breakdown. As neither direct rates of protein synthesis nor protein breakdown were measured it was not possible to delineate the relative contribution of protein synthesis vs. breakdown to the whole-body response. Moreover, tissue-specific (i.e., skeletal muscle vs. gut) turnover rates were not measured. This point is particularly relevant as the rate of gut protein turnover can be significantly higher than that of skeletal muscle (39) rendering it difficult to draw any conclusions as to whether the altered whole-body protein kinetics were a function of changes in amino acid handling at the level of skeletal muscle. What the authors did show was that omega-3 fatty acid supplementation increased the omega-3 fatty acid composition of skeletal muscle membrane phospholipids that coincided with enhanced phosphorylation of mechanistic target of rapamycin (mTOR)^Ser2448^ and ribosomal protein of 70 kDa S6 (p70S6K1)^Thr389^, two key proteins known to regulate skeletal MPS (40).

Building on the early work of Gingras et al. (38) Smith and colleagues conducted two studies in younger (17) and older (16) human adults that assessed the influence of 8 weeks of omega-3 fatty acid supplementation (1.86 g/d of EPA and 1.50 g/d DHA) on rates of mixed skeletal MPS in the fasted-state, and in response to a hyper-aminoacidemic-hyper-insulinemic infusion. These studies (16, 17) demonstrated that whilst EPA and DHA supplementation and subsequent incorporation into membrane phospholipids had no impact on fasted rates of mixed MPS, in response to the hyper-aminoacidemic-hyper-insulinemic infusion, there was a potentiation of mixed MPS compared to before supplementation. Additionally, the potentiation of mixed MPS by EPA and DHA feeding was associated with enhanced mTOR^Ser2448^ and p70S6K1^Thr389^ phosphorylation in skeletal muscle, corroborating the previous observations of Gingras et al. (38). A separate study (18), showed that 6 months of 1.86 g/d of EPA and 1.50 g/d DHA supplementation lead to a significant increase in lean mass and clinically-relevant gains in muscle volume and muscle strength in older adults in a free-living environment. When taken together with the animal work of Gingras et al. (38), these human studies (16, 17) indicated that omega-3 fatty acid intake increased the omega-3 fatty acid composition of skeletal muscle phospholipids that is linked to enhanced rates of mixed MPS supporting gains in skeletal muscle mass and size over time (18). Given that the age-related loss of skeletal muscle mass and strength with advancing age, termed sarcopenia, is now recognized as an independent condition (International Classification of Disease, ICD-10-CM) (41), the use of omega-3 fatty acids to promote skeletal muscle anabolism may soon prove to have important utility in geriatric populations.

Since the seminal investigations of Gingras et al. (38) and Smith et al. (16–18) there have been other studies examining the role of omega-3 fatty acids on skeletal muscle protein metabolism. For instance, omega-3 fatty acids have been shown to alter protein turnover in C_2_C_12_ cells (11, 12), as well as augmenting anabolic signaling in skeletal muscle of both rodents (15) and humans (33). There are also reports that supplementation with omega-3 fatty acids enhances resistance exercise-induced gains in skeletal muscle strength (42), an effect that appears to be particularly potent in older women (43). However, not all studies support the notion that omega-3 fatty acids enhance muscle anabolism. One study by McGlory et al. (44) failed to show any measurable effect of 8 weeks of 5 g/d EPA and DHA feeding on changes in myofibrillar MPS following either ingestion of 30 g protein or when protein feeding was combined with a bout of unilateral resistance exercise in young men. Additionally, Da Boit et al. (43) failed to demonstrate any effect of 2.1 g EPA/d and 0.6 g DHA/d supplementation on integrated rates of myofibrillar MPS or muscle size in older adults undergoing 18 weeks of resistance exercise training.

The conflicting reports regarding the efficacy of omega-3 fatty acid supplementation on MPS in humans could be underpinned by a number of factors, not least differences in experimental design. Unlike the repeated measures design of Smith et al. (16, 17) there was no pre-post supplementation measurement of myofibrillar MPS in the work of Da Boit et al. (43) and McGlory et al. (44) thus reducing statistical power. Furthermore, the 30 g dose of protein used by McGlory et al. (44) is a dose known to maximize rates of myofibrillar MPS in younger persons (45), whereas in the studies of Smith et al. (16, 17) amino acids were infused at a rate to elicit a state of aminoacidemia that is suboptimal for the stimulation of myofibrillar MPS. Thus, it is entirely possible that in the study of McGlory et al. (44) maximal rates of myofibrillar MPS had already been achieved and leaving no further capacity for omega-3 fatty acids to confer anabolic influence. As older adults require a greater relative per dose of protein to optimally stimulate rates of myofibrillar MPS than younger adults (0.40 vs. 0.24 g/kg body mass) (45), this contention may explain the greater relative increase in rates of mixed MPS in response to aminoacidemia in older compared to younger adults following omega-3 fatty acid feeding (16, 17). It could also provide some explanation as to the marked gains in muscle size with omega-3 fatty acid feeding in older adults in a free-living setting (18) during which dietary intake was not controlled and protein consumption likely suboptimal. Conversely, older adults who are already consuming adequate dietary protein may not receive the same benefit with omega-3 fatty acid supplementation compared to those who do not, at least with respect to changes in rates of MPS.

## Counteracting Skeletal Muscle Loss With Omega-3 Fatty Acids

### Skeletal Muscle-Disuse Atrophy

Although resistance exercise enhances rates of MPS in response to amino acid ingestion (29), periods of muscle-disuse (i.e., immobilization) result in decreased rates of MPS in both the fed and fasted state (46, 47). This reduction induces an aggregate negative state of protein balance leading to a decline in muscle mass and size over time (36). Physically active younger women also appear to be more susceptible to periods of muscle-disuse as they are ~3 times more likely to sustain anterior cruciate ligament tears in select sporting activities requiring surgical intervention compared to their male counterparts (48). Whilst younger adults recover muscle mass and size from such periods, older adults display an impaired regenerative capacity in response to episodes of muscle-disuse (49, 50). When superimposed onto the natural biological decline in muscle mass with advancing age, these periods of muscle-disuse in older adults give rise to the “catabolic crisis model” of accelerated muscle loss, rendering older persons at greater risk of premature entry to a state of functional disability (51). Strategies such as resistance exercise (52) and neuromuscular electrical stimulation (53) are effective means to attenuate muscle-disuse atrophy. However, in situations in which patients are immobilized due to surgery/injury, resistance exercise, and neuromuscular electrical stimulation may not be the most practical approaches due to associated contraindications (e.g., pain/inflammation) as well as the necessity for qualified supervision, particularly in an institutionalized setting.

Given that omega-3 fatty acid supplementation enhances amino acid and insulin-mediated increases in rates of MPS (16, 17), it is possible that omega-3 fatty acid intake may serve to attenuate disuse-induced declines in MPS and thus attenuate muscle loss. Supplementation of rodents undergoing hindlimb suspension with fish oils rich in omega-3 fatty acids has been shown to alleviate soleus atrophy, which was associated with partial preservation of myosin heavy chain content and p70S6K1^Thr389^ phosphorylation (14). Moreover, others recently demonstrated that 6-weeks of ~3 g/d EPA and ~2 g/d DHA attenuated declines in muscle volume and muscle mass during 2-weeks of unilateral leg immobilization in young women (36). A key finding of this work (36) was that following 2-weeks of free-living recovery participants in the omega-3 fatty acid group recovered the losses in muscle volume whereas those in the control group did not. The attenuation of muscle-disuse atrophy by omega-3 fatty acids also coincided with increased daily rates of integrated myofibrillar MPS. These findings (36) complement previous reports using amino acid and insulin infusions (16, 17) and highlight the efficacy of omega-3 fatty acid feeding to protect the loss of skeletal muscle in response to, and recovery from, periods of muscle-disuse in young women. It is important to note that this study (36) was conducted in the context of simple muscle-disuse atrophy and in the absence of factors that likely accompany injury/recovery from surgery such as excessive inflammation/hypercortisolemia. It is also unknown whether omega-3 fatty acid feeding protects muscle loss during periods of muscle-disuse in older men and women or younger men. Further work in situations that recapitulate real-life clinical scenarios of muscle-disuse in both younger and older adults would add to these findings.

### Cancer Cachexia

**Figure 1:**
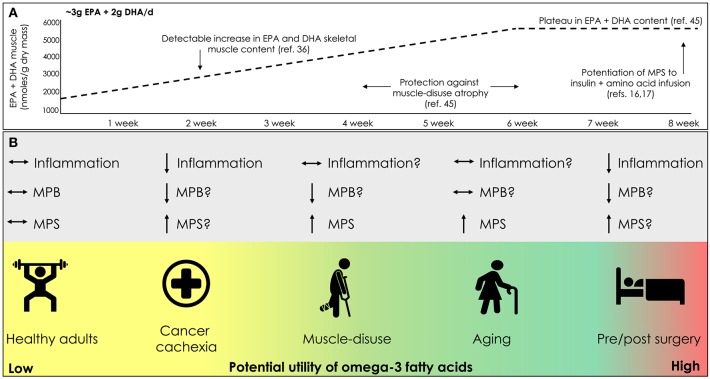
**(A)** Time course change in skeletal muscle lipid content with omega-3 fatty acid supplementation. **(B)** Potential clinical scenarios for the use of omega-3 fatty acid supplementation to promote and/or mitigate losses in skeletal muscle mass; eicosapentaenoic acid (EPA), docosahexaenoic acid (DHA), muscle protein synthesis (MPS), muscle protein breakdown (MPB).

As omega-3 fatty acid intake has been shown to confer anabolic influence in ostensibly healthy individuals, it is entirely possible that omega-3 fatty acid intake may also positively impact skeletal muscle in situations of disease (Figure 1). Cancer cachexia is a multifactorial syndrome characterized by a marked involuntary loss of skeletal muscle mass that has a negative impact on muscle function, and is highly predictive of poor survival (54). Treatment of cancer cachexia continues to be one of the most prominent challenges faced by clinicians and scientists since the beginning of modern cancer therapy (55). The lack of adequate energy and nutrient ingestion, high concentration of plasma pro-inflammatory factors, tumoral factors, chemo/radiotherapy, and low-physical activity all contribute to the loss of muscle mass seen with cancer cachexia (55–58). As such, in the clinical setting a multifactorial approach that includes increased physical activity and targeted nutritional strategies is often employed to combat cancer cachexia.

Most nutritional guidelines targeted at attenuating cancer cachexia focus on reaching energy requirements of 25–30 kcal/kg/day and protein ingestion of 1.2–1.5 g/kg body mass/day (59, 60). Due to complications associated with some types of cancers (e.g., esophageal) and related surgeries resulting in dysphagia, achieving these guidelines in a real-world scenario can be problematic. Omega-3 fatty acids, mainly EPA (at 2–2.5 g/day) (59, 60) have been given as part of the anti-inflammatory and anti-catabolic nutritional therapy to combat the pro-inflammatory burden of cancer cachexia (58). The use of omega-3 fatty acids to counteract cancer cachexia came to practice following studies in rodents with various types of cancer showing that ingestion of fish oils rich in EPA and DHA (61–63) or increasing the omega-3 to omega-6 fatty acid ratio in the diet (64) were effective in decreasing tumor growth and cachexia development. After these initial studies (61–63), further reports were published in humans corroborating the positive effects of omega-3 fatty acids seen in rodent models. For example, there is evidence that low skeletal muscle mass is associated with reduced plasma fatty acid EPA status in cancer patients (65). Provision of 2.2 g/d EPA for 5 d pre-operatively and 21 d postoperatively in patients undergoing esophageal cancer surgery has been shown to preserve lean body mass as assessed by bioelectrical impedance (66). Furthermore, others have shown in patients with mixed-stage non-small cell lung cancer that 2.5 g/d of EPA + DHA resulted in a significant gain in lean body mass and a corresponding decrease in fat mass (67). However, the experimental evidence supporting the use of omega-3 fatty acids in the treatment of cancer cachexia is far from conclusive. A recent systematic review of studies published from 2000 to 2015 examining the impact of omega-3 fatty acids on cancer cachexia identified that out of 140 studies only 7 reached the quality threshold of inclusion according to the Delphi list (68). Out of those 7 studies, only one study in pre-cachexic cancer patients demonstrated a statistically positive effect of omega-3 fatty acids (69, 70). The fact that only 5% of available studies reached the required threshold of quality in this systematic review (68), highlights the challenges faced by scientists and clinicians in conducting high-quality, statistically-powered, randomized controlled trials in this specialized population.

Unlike periods of uncomplicated muscle-disuse in which the declines in skeletal muscle mass are primarily driven by a decrement in rates of MPS (71), it is generally assumed that cachexia is underpinned by both a diminished rate of MPS and an elevated rate of protein breakdown induced by a hyper-inflammatory state (Table 1). Given the anti-inflammatory effects of omega-3 fatty acids (see section Anti-inflammatory Effects of Omega-3 Fatty Acids) taken together with the stimulatory influence of omega-3 fatty acids on MPS (16, 17), it is likely that any impact of omega-3 fatty acids on lean body mass in cancer cachexia is a result of the dual action on both MPS and protein breakdown. To our knowledge, no study has directly assessed the impact of omega-3 fatty acids in isolation on changes in rates of MPS or protein breakdown in human cancer patients. One study in patients with various types of cancer (i.e., lung, colorectal, breast, esophagus, b-cell lymphoma) demonstrated that ingestion of a multi nutritional supplement containing 11 g whey protein, 4 g leucine, and 2.2 g EPA and 1.1 g DHA increased MPS above control (75). Whether the inclusion of omega-3 fatty acids in this formula was additive toward rates of MPS is unknown. However, it is unlikely that omega-3 fatty acids contributed to the enhanced MPS response given that MPS was measured 5 h post supplement ingestion and omega-3 fatty acids at the dose provided, would not have been incorporated into skeletal muscle within such a time-frame (33). Due to ethical limitations associated with multi-biopsy sampling in cancer cachexic patients, data related to muscle protein turnover in this clinical population are sparse. The introduction of the “virtual biopsy” procedure in which the synthetic rate of plasma proteins is used as a proxy of muscle proteins (79), may serve to circumvent ethical issues related to biopsy sampling and contribute to the development of nutritional interventions to combat cancer cachexia. However, more work is needed to validate this approach in compromised populations particularly during conditions of inactivity and muscle atrophy.

**Table 1:** Skeletal muscle protein synthesis and breakdown rates in patients with cancer cahchexia.

**References**	**Methods**	**Basal MPS controls**	**Type of cancer**	**Basal MPS cancer**	**Postprandial MPS cancer**	**Basal MPB cancer**	**Nutritional intervention**
Emery et al. (72)	Primed infusion [^13^C_2_]-Leu and ^13^C labeled sodium bicarbonate and continuous [^13^C_2_]-Leu	0.198 ± 0.020 (%h)	Kidney and lung cancer (pre-treatment)	0.030 ± 0.007 (%/h)	−	−	−
Dworzak et al. (73)	Primed L-[^2^H_5_] phenylalanine and L[^2^H_4_] Tyrosine and continuous -[^2^H_5_] phenylalanine	0.048 ± 0.013 (%/h)	Advanced gastric carcinoma (pre-treatment)	0.021 ± 0.004 (%/h)	−	−	−
Dillon et al. (74)	Primed continuous infusion L-[ring-^2^H_5_]-Phe	−	Ovarian cancer (during treatment)	0.052 ± 0.009 (%/h)	0.120 ± 0.008 (%h)	−	Amino acid supplement
Deutz et al. (75)	Primed continuous infusion L-[ring-^13^C_6_]-Phe	−	Lung, colorectal, Breast, Esophagus, b-cell Lymphoma (no treatment for 4 weeks before the study)	0.073 ± 0.023 (%/h) 0.073 ± 0.022 (%/h)	0.065 ± 0.028 (%h) 0.097 ± 0.033 (%h)	−−	Conventional medical food Re-designed medical food
Dillon et al. (76)	Pulse bolus injection L-[ring-^13^C_6_]-Phe and 15N-Phe	−	Recurrent cervical carcinoma (case study)	0.07 (%/h)	−	0.03 (%/h)	−
Williams et al. (77)	Primed continuous infusion [1,2-^13^C_2_]-Leu and ring-D5-Phe	0.038 (%h)	Colonic adenocarcinoma booked for curative resection	0.028 ± 0.004 (%/h)	0.038 ± 0.004 (%/h)	−	Intravenous mixed amino acids
MacDonald et al. (78)	Single dose Deuterium oxide 133 g (70 Atom %)	37.2 [34.0–45.4] (g/day)	Upper gastrointestinal cancer	41.1 [38.2–41.8] (g/day)		42.4 [39.1–42–8] (g/day)^a^	−

*MPS, muscle protein synthesis; MPB, muscle protein breakdown; Leu, leucine; Phe, phenylalanine*.

a *Calculated indirectly based on muscle mass loss*.

## Mechanisms of Action of Omega-3 Fatty Acids

**Figure 2:**
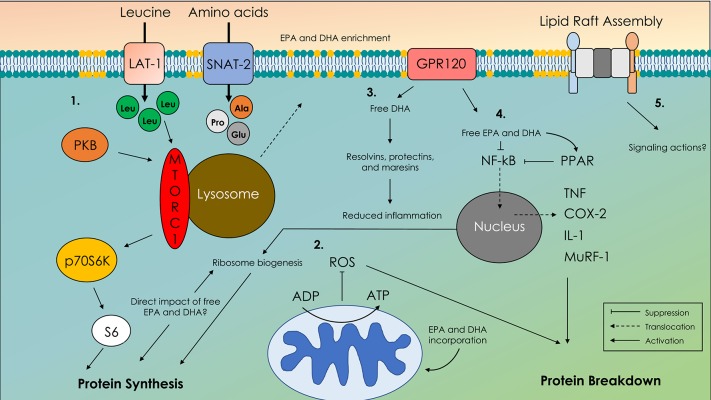
Schematic illustration of molecular mechanisms of action of omega-3 fatty acids in skeletal muscle. 1. Translocation of the mechanistic target of rapamycin complex-1 (mTORC-1) with the lysosome to the membrane in close proximity to amino acid transporters. 2. Enhanced adenosine diphosphate (ADP) sensitivity and altered reactive oxygen species emissions (ROS). 3. G-coupled protein receptor 120 (GPR120) and free docosahexaenoic acid (DHA)-mediated production of resolvins, protectins, and maresins. 4. Cystolic retention of nuclear factor kappa B (NF-κB) preventing upregulation of proteolytic and pro-inflammatory agents. 5. Altered lipid raft formation that acts as signaling platforms for unknown signaling agents; eicosapentaenoic acid (EPA), docosahexaenoic acid (DHA).

Traditional thought is that their anti-inflammatory properties are primarily responsible for many of the reported health benefits of omega-3 fatty acids (1). In diseased states that are often accompanied by a state of excessive inflammation, the production of anti-inflammatory molecules and corresponding suppression of pro–inflammatory agents induced by omega-3 fatty acids is thought to underpin improved health status (1). However, in healthy adults, reports of enhanced MPS (16) and increased muscle mass (18) with omega-3 fatty acid feeding occurred in the absence of any corresponding change in the concentration of putative circulating inflammatory markers. These findings (16, 18) suggest that in non-pathological states, omega-3 fatty acids do not confer anabolic influence via an anti-inflammatory mechanism. A schematic illustration of the potential actions of omega-3 fatty acids in skeletal muscle addressed in the following sections can be seen in Figure 2.

### EPA vs. DHA

Whilst studies often provide EPA and DHA in combination, both fatty acids are known to exert independent biological actions. Reports have shown that EPA may have a greater influence on muscle protein turnover (11, 12) whereas DHA, likely owing to its higher content in neuromuscular tissues [~50 times higher than EPA in brain (80)], is heavily involved in neuromuscular function (81). Work in C_2_C_12_ myotubes has demonstrated that treatment with 50 μM EPA but not 50 μM DHA stimulated an increase in protein synthesis (11) and a decrease in protein breakdown (11). Others, again in C_2_C_12_ myotubes, have shown that 24 h incubation with 50 μM EPA resulted in protein accretion, an effect likely driven by decreased protein breakdown, with no effect of 50 μM DHA. Although delineating the differential effects of EPA and DHA on muscle protein turnover *in vitro* is interesting, the concentration of fatty acids used in each experiment may have a direct bearing on the outcome (23, 82). Indeed, in one report, treatment of C_2_C_12_ myotubes with 400–600 μM of EPA resulted in a decrease in protein degradation; however, a similar effect on protein degradation was also achieved across a range of 300–700 μM DHA (82). Importantly, the concentrations of EPA and DHA used in many *in vitro* studies are higher than would typically be seen in the human bloodstream even after high-dose supplementation (83). To our knowledge, no study has directly compared the effect of physiological doses of EPA vs. DHA ingestion on rates of MPS or protein breakdown in humans. Given that EPA and DHA serve as the substrates for the production of different pro-and anti-inflammatory mediators (e.g., resolvins) each with their own specialized function (1), defining the mechanisms underpinning how EPA and DHA alter muscle protein turnover in an *in vivo* setting is an interesting area worthy of future work.

### Amino Acid Transport

As omega-3 fatty acids appear to promote anabolism via enhanced feeding-induced increases in MPS (16, 17), one potential mechanism by which omega-3 fatty acids alter rates of MPS could be that of enhanced amino acid transport. One study in pigs (84) identified an increase in the mRNA expression of the system L-amino acid transporter (LAT-1) following the ingestion of a diet rich in omega-3 fatty acids. As LAT-1 is known to transport the amino acid leucine, which is a key agonist of MPS, it could be contended that omega-3 fatty acid modulation of the phospholipid membrane somehow enhances LAT-1 expression thus facilitating leucine-mediated stimulation of MPS. This theory may, in part, explain the observation of enhanced rates of MPS in response to a hyper-aminoacidemic hyper-insulinemic infusion (16, 17). There is some experimental evidence for this thesis in humans, as in response to immobilization omega-3 fatty acid feeding has been shown to increase the LAT-1 mRNA expression in young women, which was linked to higher integrated rates of MPS (6). However, the change in LAT-1 mRNA expression in that study (36) did not translate into a detectable increase in LAT-1 protein content. Whether omega-3 fatty acid feeding alters the expression and/or function of other amino acid transporters remains unknown.

### Protein Kinase Activity

Many studies that have shown a positive influence of omega-3 fatty acids on skeletal muscle anabolism also detect increases in the phosphorylation status of kinases related to the mTORC-1 signaling axis (e.g., protein kinase B (PKB)^Thr308/Ser473^, mTOR^Ser2448^, p70S6K1^Thr389^) (11, 15–17). There is also evidence that omega-3 fatty acid supplementation increases the content of mechanically-sensitive protein kinases upstream of mTORC-1 (33). These findings would be expected as the mTORC-1 signaling axis is important for acute nutrient- and contraction-mediated increases in rates of MPS in humans (40, 85). However, using radiolabelled (γ-^32^P) ATP, providing as a gold-standard measurement of protein-kinase activity *in vitro*, we identified a suppression of p70S6K1 activity in response to protein feeding and resistance exercise following 8 weeks of omega-3 fatty acid intake in young men (86). There was even a downregulation in the activity of PKB in response to omega-3 fatty acid supplementation alone. One consideration is the *in vitro* kinase assay is a V_max_ measure of kinase activity and does not necessarily reflect *in vivo* kinase function, nor single-residue phosphorylation status. It is also entirely possible that omega-3 fatty acids influence muscle anabolism via mTORC-1 independent mechanisms. Indeed, the study in which 50 μM EPA but not 50 μM DHA stimulated an increase in protein synthesis in C_2_C_12_ myotubes also identified an increase in the phosphorylation of p70S6K1^Thr389^ with both EPA and DHA (11) suggesting EPA stimulates protein synthesis via alternative or additional mechanisms to mTORC1-p70S6K1 signaling (87). There is evidence that omega-3 fatty acids act via mitogen-activated protein kinase (MAPK) signaling and/or alterations in satellite cell activity, which could have important implications for muscle regeneration in aging and in recovery from exercise; for an extended review see (23). Although there is little evidence that MAPK signaling and satellite cell activity play any significant role in mediating acute feeding-induced increases on rates of MPS and it is more likely that other, potentially unknown kinases or at least those not typically associated with mTORC-1 signaling, mediate the response.

Another potential mechanism mediating the potentiation of MPS with simulated feeding (16, 17) could be that of changes in mTORC1-lysosomal interactions. Recent work using immunohistochemical approaches has demonstrated that mTOR localization to lysosomal and cell membranes is a key step in mTORC-1 activation (88), and presumably MPS in response to amino acid provision. Whether omega-3 fatty acid feeding affects these processes remains unknown, but given that incorporation of omega-3 fatty acids into lipid membranes alters membrane-associated proteomic profiles (12), future work utilizing a combination of immunohistochemical and immunoprecipitation approaches coupled with direct measurement of muscle protein turnover would provide further insight.

### Mitochondrial Function

In addition to the sarcolemma, mitochondrial membranes are known to be sensitive to omega-3 fatty acid intake (37). One study has demonstrated that 12 weeks of omega-3 fatty acid supplementation (3 g EPA + 2 g DHA daily) increased mitochondrial EPA and DHA content in young men that was concordant with improved ADP sensitivity (5). Similarly, others have shown that 8 weeks of omega-3 fatty acid-rich tuna supplementation reduced whole-body oxygen consumption during steady-state exercise (89). Although improved respiration kinetics with omega-3 fatty acids are unlikely to explain previous reports of enhanced rates of MPS in response to feeding (16, 17), there is evidence that omega-3 fatty acid-mediated changes in mitochondrial function may play a role in mitigating muscle loss during aging and periods of muscle-disuse. Indeed, in the work of Smith et al. (18) in which 6 months of 1.86 g/d of EPA and 1.50 g/d DHA supplementation promoted gains in muscle size in older adults, there was also a corresponding increase in the expression of mitochondrial-related transcripts (90). Moreover, it was recently shown that the alleviation of muscle loss during 2 weeks of unilateral limb immobilization in young women undergoing omega-3 fatty acid supplementation (36) was linked to the preservation of maximal and submaximal ADP sensitivity as well as mitochondrial protein content (6). This is an important point, as ADP-stimulated oxidative phosphorylation reduces reactive oxygen species (ROS) emission, and aberrant ROS have been implicated in the pathology of muscle-disuse atrophy (91). Thus, collectively, these data (6, 36) suggest that the preservation of mitochondrial function plays a key role in the regulation of muscle size during periods of muscle-disuse in young women, which may be alleviated by omega-3 fatty acid supplementation. However, in that study (6, 36), immobilization did not alter H_2_O_2_ emissions in either the omega-3 fatty acid group or control group indicating that the mechanisms by which omega-3 fatty acids protect against muscle disuse atrophy at least in young women are unrelated to ROS emissions and oxidative stress. More work is now needed that provides insight into the interaction between mitochondria, omega-3 fatty acids, and rates of MPS in skeletal muscle.

### Anti-inflammatory Effects of Omega-3 Fatty Acids

Diseased states such as cancer cachexia are associated with increased expression of pro-inflammatory cytokines (e.g., IL-1, IL-6, and TNF) and acute phase proteins (e.g., CRP). These inflammatory markers are known to trigger regulators of proteolysis that in turn promote muscle loss (92, 93). The classic mechanism of action by which EPA and DHA modify the production of pro-inflammatory cytokines is through alteration in the synthesis of lipid mediators, principally derivatives of the omega-6 fatty acid arachidonic acid (ARA) and of EPA and DHA themselves. These lipid mediators are biologically active and include prostaglandins and leukotrienes as well as specialized pro-resolution mediators. The fatty acid substrate (e.g., ARA, EPA or DHA) for production of lipid mediators is released from cell membrane phospholipids through the action of phospholipase enzymes, in particular phospholipase A2. Typically, ARA is more abundant than EPA or DHA [i.e., it is reported to comprise 10.5% of fatty acids in skeletal muscle lipids (33) and 17.2% of fatty acids in skeletal muscle phospholipids (17), and therefore it is the dominant substrate]. ARA is metabolized by cyclooxygenase (COX) enzymes (e.g., COX-2) to 2-series prostaglandins and by 5-lipoxygenase (LOX) to 4-series leukotrienes. These mediators are closely involved in inflammatory processes, acting through specific G-protein coupled receptors. Enrichment of EPA in cell membranes is partly at the expense of ARA, thus altering the balance of substrates available. This is seen in both inflammatory cells (94) and in skeletal muscle (17, 33). EPA is also metabolized by COX and LOX enzymes but gives rise to metabolites with a slightly different structure from those produced from ARA, typically resulting in lower affinity for receptors (95) and lower bioactivity (94). As a result, EPA enrichment is linked with lower concentrations of potent ARA-derived mediators being produced and higher concentrations of less potent EPA-derived mediators being produced. EPA and DHA can also decrease COX-2 gene and protein expression (62, 96), which has the effect of lowering lipid mediator production due to less available enzyme.

The mechanism behind the omega-3 fatty acid-induced lowering of COX-2 gene expression seems to be inhibition of the nuclear factor kappa B (NF-κB) pathway. NF-κB is a transcription factor that acts to up-regulate inflammatory gene expression (97). NF-κB exists as an inactive trimer in the cytosol of cells. In the presence of an inflammatory trigger or stimulus, a signaling pathway results in phosphorylation of the inhibitory subunit of the NF-κB trimer which then dissociates and is degraded. This leaves the remaining dimer free to translocate to the nucleus and bind to response elements in target genes altering their transcription. Through inhibiting the signaling pathway that activates NF-κB, EPA, and DHA not only down-regulate COX-2 gene expression but also the expression of genes encoding common pro-inflammatory cytokines like TNF and IL-1, genes encoding important chemokines like monocyte chemotactic protein-1, and genes encoding adhesion molecules responsible for leukocyte infiltration (94). The inhibition of NF-κB activation by EPA and DHA is linked to changes in cell membranes (98, 99) suggesting omega-3 fatty acid induces alterations in very early signaling events. In addition, EPA and DHA and some of their lipid mediator derivatives can activate peroxisome proliferator-activated receptor (PPAR) γ (100, 101), which physically interferes with NF-κB translocation to the nucleus (102). Consistent with the importance of this interaction, knockdown of PPARγ significantly reduced the effect of EPA on NF-κB signaling (103). Another target for NF-κB is the muscle ring finger-1 (MuRF-1) gene (103) linking this pro-inflammatory pathway directly with muscle protein breakdown as MuRF-1 aids protein degradation through the ubiquitination pathway (104).

It appears that EPA and DHA can down-regulate NF-κB activation through several mechanisms, one being through activation of PPARγ (100), a second being action via a G-protein coupled receptor GPR120 (105), and a third being through effects within the cell membrane (98, 99). GPR120 was first identified to be expressed on inflammatory macrophages and adipocytes, but has more recently been described on skeletal muscle cells (106). DHA appears to be the major endogenous ligand for GPR120 and DHA was shown to inhibit NF-κB activation and expression of NF-κB target genes and proteins via GPR120 (105). GPR120 was also involved in beneficial metabolic effects of DHA in adipocytes (105) and skeletal muscle (106), but whether GPR120 mediates anti-inflammatory effects of DHA in skeletal muscle has not been reported.

The effects of DHA on NF-κB activation and NF-κB mediated events have been shown to involve modifications to cell membrane structures termed lipid rafts (99). Lipid rafts are cell membrane regions that are rich in sphingolipids, saturated fatty acids, cholesterol and signaling proteins. They form in response to certain stimuli and act to bring together different proteins involved in common signaling pathways, essentially forming signaling platforms. Lipid rafts are well described in immune cells, cancer cells and neurones. They are also described in skeletal muscle (107) and intriguingly they are disrupted by short term muscle disuse in the rat (108). Some saturated fatty acids have been shown to promote lipid raft formation and inflammatory signaling (98, 99) while DHA was shown to inhibit lipid raft formation in response to inflammatory stimuli, including saturated fatty acids, and this was linked to reduced activation of the NF-κB pathway (98, 99). It is not known if omega-3 fatty acids affect lipid raft formation in skeletal muscle cells and whether such an effect might be linked to reduced inflammation and the expression of molecules that regulate muscle protein turnover.

The effects of EPA and DHA on production of prostaglandins and leukotrienes and on pathways that reduce NF-κB activation and subsequent production of pro-inflammatory cytokines, chemokines and adhesion molecules are generally regarded as being anti-inflammatory (1, 94). It is now known that EPA and DHA are substrates for lipid mediators that actively turn-off (i.e., resolve) inflammation (109–111). These so-called specialized pro-resolution mediators include resolvins produced from EPA (E-series) and DHA (D-series) and protectins and maresins produced from DHA. The synthesis of resolvins, protectins, and maresins involves the COX and LOX pathways, with different epimers being produced in the presence and absence of aspirin (109–111). As might be expected, resolvin synthesis is increased by feeding laboratory rodent diets rich in EPA and DHA (112) and there are reports of increased levels of various resolvins in human serum and plasma following daily intake of omega-3 fatty acid supplements for a period of weeks (113, 114). The biological effects of resolvins, protectins and maresins have been examined extensively in cell culture and animal models of inflammation, and they have been demonstrated to be anti-inflammatory and inflammation resolving, preventing leukocyte infiltration into tissue and decreasing production of cytokines like TNF and IL-1β (109–111). A recent study (115) mapped the lipid mediator signature during a murine model of muscle injury and regeneration and identified a temporal pattern of production of classic pro-inflammatory mediators like prostaglandins/leukotrienes and pro-resolving mediators like resolvins. These mediators were produced by infiltrating leukocytes (neutrophils and macrophages) and the temporal change was linked to a change in phenotype of these leukocytes. The resolution phase was associated with the emergence of an anti-inflammatory phenotype of macrophage. The role of such lipid mediators in muscle protein turnover and how this may be optimized by managing omega-3 fatty acid exposure is not currently known.

## Future Directions and Conclusion

In summary, the available evidence would suggest that omega-3 fatty acid intake has the potential to enhance skeletal muscle anabolism, but the magnitude of the effect may be dependent upon a number of factors. These factors include, but are not limited to, the daily dose of protein intake, measurement technique, as well as age and metabolic status of participants. One particular area of promise is the potential for omega-3 fatty acids to counteract muscle atrophy, and promote recovery, from periods of muscle-disuse induced by surgery and subsequent bedrest/inactivity. However, before firm conclusions can be drawn as to the efficacy of omega-3 fatty acid intake on musculoskeletal health and subsequent translation to the clinical setting there remains many unanswered questions that require experimental attention. For instance, what are the molecular mechanisms that mediate improved skeletal muscle protein turnover and functionality with omega-3 fatty acid intake? Is there a dose-response relationship between omega-3 fatty acid intake and physiological outcomes, and is the efficacy of omega-3 fatty acid intake on skeletal muscle influenced by sex? Given their independent biological actions, it will also be vitally important to discern the independent roles of EPA and DHA in mediating changes in skeletal muscle plasticity. Another important but often overlooked factor is what are the off-target effects of increasing omega-3 fatty acid intake and are there any negative consequences in other vitally important processes. The answers to such questions will inevitably require the application of a range of invasive and non-invasive methodologies in pre-clinical models as well as humans. We hope that such work will provide important information for the development of omega-3 fatty acid therapies to promote musculoskeletal health in a variety of settings and populations.

## Author Contributions

CM, PC, and EN contributed to the writing and critical evaluation of the manuscript. All authors approved the final version of the manuscript for submission.

### Conflict of Interest Statement

The authors declare that the research was conducted in the absence of any commercial or financial relationships that could be construed as a potential conflict of interest.
